# A Speed-Dependent Assessment of E-Textile-Based Sensor Technology: Validity of the Prevayl Wearable Heart Rate Monitor

**DOI:** 10.3390/s26113378

**Published:** 2026-05-26

**Authors:** Louise C. Burgess, Matthew Armstrong, Louise Beresford, Andrew J. Callaway

**Affiliations:** 1 School of Allied Health and Exercise Science, Bournemouth University, Poole BH12 5BB, UK; lburgess@bournemouth.ac.uk (L.C.B.); matthew.g.armstrong@durham.ac.uk (M.A.); lberesford@bournemouth.ac.uk (L.B.); 2 Department of Sport and Exercise Sciences, Durham University, Durham DH1 3LA, UK

**Keywords:** validity, reliability, wearable technology, e-textiles, heart rate, ECG

## Abstract

**Highlights:**

**What are the main findings?**

- The Prevayl garment-based heart rate monitor demonstrated strong validity against 3-lead ECG during incremental running, achieving correlation coefficients of r = 0.94 for raw beat count and r = 0.96 for the device algorithm.
- The device maintained consistent accuracy across all running speeds (0–12 kph) with 100.5% median agreement to ECG and negligible systematic bias of 0.04 beats in Bland–Altman analysis.

**What are the implications of the main findings?**

- E-textile garment-integrated sensors provide a valid and reliable alternative to traditional chest strap heart rate monitors for continuous cardiovascular monitoring during dynamic exercise.
- Athletes, coaches, and sports scientists can confidently use the Prevayl system for accurate heart rate monitoring across varied exercise intensities, supporting evidence-based training and performance optimisation.

**Abstract:**

Background: The use of wearable sensors to measure and monitor heart rate has exponentially grown in recent years, representing an inexpensive, time-efficient, and non-invasive method to assess the status of cardiovascular fitness and the autonomic nervous system. Validating new devices against a criterion standard, such as electrocardiography (ECG), is essential to ensure their accuracy and reliability. This study examined the accuracy and validity of the Prevayl heart rate monitor against 3-lead ECG. Methods: Twenty-six healthy adults (15 female, mean age 32.0 ± 10.4 years) completed a 16-min, incremental running test on a treadmill. Heart rate data were recorded simultaneously throughout the test via ECG and the Prevayl wearable and compared retrospectively. Beat count error (%), mean heart rate absolute error (beats per minute (bpm)), and percentage error (bpm) were calculated. In addition, a Bland–Altman analysis and Pearson’s correlation coefficient were conducted to assess agreement and correlation, respectively. Results: The Prevayl device demonstrated a median beat count agreement of 100.5% with ECG (range: 98.6–104.4%; N_part_ = 26). Strong correlations were observed between ECG and Prevayl for both raw beat count (*r* = 0.94, *p* < 0.01) and heart rate (beats per minute (bpm)) from ECG and the Prevayl algorithm (*r* = 0.96, *p* < 0.01). Across running speeds (0–12 kph), a strong correlation was found between raw beat count from ECG and Prevayl (*r* = 0.82–0.89, *p* < 0.01) and between bpm from ECG and Prevayl (*r* = 0.86–0.93, *p* < 0.01). Bland–Altman plots demonstrated negligible systematic bias. Conclusions: The Prevayl system provides valid measurements when compared to ECG during incremental running. This is demonstrated through strong correlations to ECG heart rate data at different speeds and with different analysis methods, supporting its use for monitoring cardiovascular responses during exercise.

## 1. Introduction

The use of wearable sensors (i.e., smart watches and bands) to measure and monitor heart rate has exponentially grown in recent years, representing an inexpensive, time-efficient, and non-invasive approach for assessing cardiovascular fitness and autonomic nervous system activity [1]. These devices typically operate via one of two mechanisms: chest-worn sensors that detect cardiac electrical signals and relay them to an external receiver (such as a watch or smartphone app) or wrist-worn monitors that derive heart rate from photoplethysmography by detecting changes in blood volume [2]. Evidence suggests that chest-worn devices generally demonstrate superior accuracy compared to wrist-worn devices, as they capture the heart’s electrical activity directly from the thorax, closer to the source, and benefit from more stable electrode–skin contact [3]. Regardless of device, the effectiveness of continuous heart rate monitoring remains dependent on the ability of wearable sensors to provide valid and reliable measurements [4].

To ensure the validity and reliability of wearable sensors, new devices should be validated against either the criterion measure (electrocardiogram (ECG)) or a previously validated heart rate device [5]. ECG continues to be considered the gold standard measure of heart rate across all forms of health and sports performance [6]. The detection of ECG signals is used to determine heart rate from either R-R intervals (expressed as the length of the interval between QRS complexes) or by counting the number of R peaks (beats) across a specific time frame [7]. Typically, chest-worn heart rate devices verified against ECG have achieved a correlation coefficient of r > 0.90 and a standard error of estimate within 5 beats per minute (bpm) of an ECG during rest, recovery, and varying exercise intensities [8]. A recent systematic review included 32 studies across clinical, sporting, or occupational settings that had evaluated the use of chest-worn heart rate monitors against ECG or another garment [3]. The highest performing device in terms of heart rate measurement accuracy was the Polar chest strap models (H7 and H10), with a correlation coefficient of 0.99 between the Polar H7 and reference values [9]. The review concluded that chest-worn devices represent a reliable and versatile tool for real-time cardiac monitoring, although it highlighted the lack of data at sub-maximal and maximal exercise intensities [3]. Similarly, Carrier et al. (2020) confirmed that most studies in this area are evaluated at a self-selected pace rather than during incremental exercise to reflect the demand of the devices in practice [10].

Advances in e-textile solutions, where sensors are embedded directly into fabrics rather than housed in separate straps, have enabled further innovation in wearable monitoring devices [11]. However, these developments necessitate careful consideration of the validity and reliability of data obtained [11]. A 2020 review of 16 smart garments (three commercially available and 13 working prototypes) reported that t-shirts with integrated heart rate monitoring demonstrate acceptable validity at rest and during sub-maximal exercise, although accuracy decreases with exercise intensity [12]. For example, an evaluation of the Hexoskin biometric shirt found correlations of 0.99 at rest, 0.91 at sub-maximal exercise intensity, and 0.90 at maximal exercise intensity when compared to heart rate data recorded via ECG [13]. Similarly, Navalta et al. (2020) evaluated three sports bras during rest and self-paced walking and found the Adidas Smart sports bra was only valid at rest (ICC = 0.79), the Sensoria biometric bra was valid during rest and walking (ICC = 0.96), and the Berlei sports bra was valid during rest, walking, and self-paced running (ICC = 0.99) when compared to the Polar H7 heart rate monitor [14]. Khundaqji et al. 2020 also highlighted a notable delay between the publication of prototype design studies and subsequent validation research, suggesting the need for ongoing validation of new garments to ensure the accuracy of the data they provide [12]. It has been reported that around 50% of new wearable sensors have not received independent validity against another measure of heart rate [15]. The data processing algorithms of the raw data are often proprietary information, unique to each device, where such processing algorithms may or may not account for dropped beats due to sensor movement. Therefore, it is important that new wearable sensors are correctly validated, with clear utilisation required not only at rest but also during different intensities of exercise and recovery, to reflect their use in practice.

This study aimed to determine the accuracy and validity of the Prevayl heart rate monitor (Prevayl Limited, Manchester, UK) against a 3-Lead ECG (Mega Electronics Ltd., Pioneerinkatu, Finland), representing the first investigation to directly compare these systems. The objectives of the study were to (i) compare heart rate derived from the reference ECG signal with heart rate derived from the Prevayl raw ECG signal and (ii) compare heart rate derived from the reference ECG signal with heart rate generated by the Prevayl proprietary algorithm during an incremental running test. This approach was designed to expand the evidence base in a condition that remains underrepresented in the existing literature, despite its relevance to applied monitoring.

## 2. Materials and Methods

### 2.1. Study Design and Participants

This validation study was conducted in the School for Allied Health and Exercise Science at Bournemouth University in the United Kingdom (UK). The protocol was approved by the institutional research ethics committee [ID: 46540]. Participants were recruited from the local area through online advertisement (X, University social media channels). Participants were eligible to take part if they were (i) 18 years or older; (ii) physically active and capable of running for 16 min on a treadmill without a break; and (iii) capable of running at a speed of 12 kph for at least 2 min. Participants were excluded if they had previous heart surgery or a known heart condition. Written informed consent was provided by all participants prior to the experiment commencing. Demographics of these participants are shown in Table 1.

### 2.2. Sample Size

An a priori power analysis was conducted using G*Power version 3.1.9.6 (9) to estimate the required sample size for correlation analysis. To detect a medium effect size (r = 0.50) with 80% power and a significance level of α = 0.05 (two-tailed), the required minimum sample size was calculated as N_part_ = 29. The study successfully recruited N_part_ = 26 participants. A post hoc power analysis was conducted to evaluate the statistical power achieved with the final sample, using the observed effect sizes. For a correlation of r = 0.94 (raw beat count) and r = 0.96 (Prevayl algorithm bpm), the achieved power exceeded 99.9%, confirming that the sample size provided sufficient statistical sensitivity for the main outcome measures.

It is important to note that the analysis included multiple statistical techniques beyond correlation, such as Bland–Altman plots and agreement metrics (e.g., beat count error, percentage, and absolute error). While specific power calculations for Bland–Altman analysis are not standardised, the high number of data pairs (N_win_ = 6248) across participants provides robust statistical sensitivity. Similarly, descriptive metrics such as beat count error and absolute and percentage error values were not tested with hypothesis-based statistics, and thus formal power calculations for those outcomes were not considered.

### 2.3. Equipment

To measure ECG, the Faros 180° device (Mega Electronics Ltd., Pioneerinkatu, Kuopio, Finland) was used, recording at 500 Hz and attached to participants using three pre-gelled disposable electrodes (Ambu VLC-00-S/25, Ambu GmbH, Bad Nauheim, Germany). The American Heart Association highlights that 3-lead ECGs are often used in exercise testing protocols where continuous monitoring is required throughout dynamic activity [1]. One electrode was placed just below the right clavicle with the Faros device attached, one just below the left clavicle, and one on the left side of the chest below the 12th rib.

**Figure 1:**
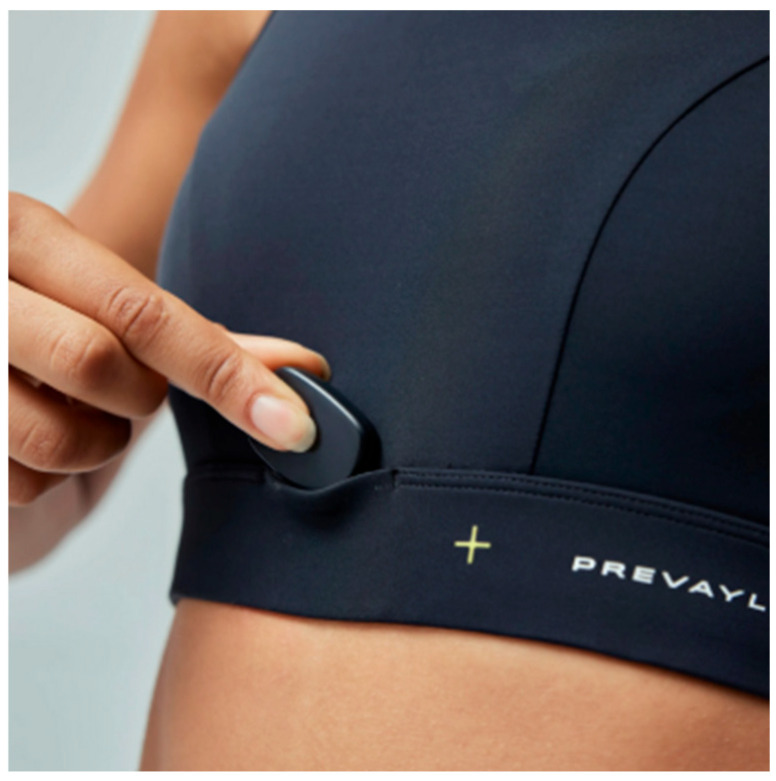
Prevayl sports bra.

**Figure 2:**
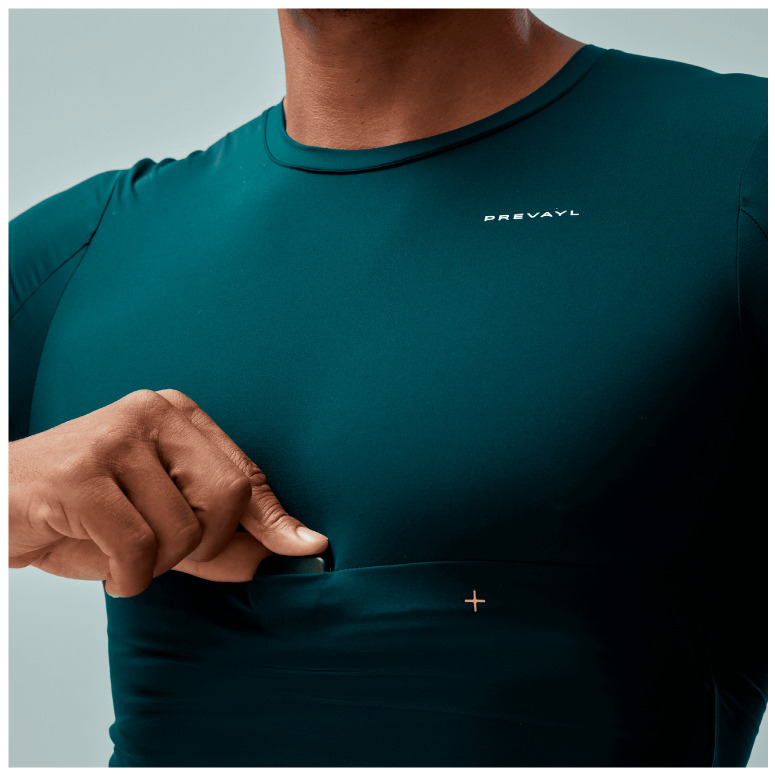
Prevayl t-shirt.

Prevayl (Prevayl Ltd., Manchester, UK) garments consisted of either a sports bra (females) (Figure 1) or a t-shirt (males) (Figure 2). The garment incorporates conductive textile-based electrodes embedded within the fabric [16], which are positioned over the thoracic region to approximate a single-lead ECG configuration. The system utilises dry electrodes, relying on fabric compression and direct skin contact to acquire electrical signals generated by cardiac depolarisation. Garment sizes were chosen by the participants and verified by the research team by checking for movement and contact pressure to ensure the sensor made consistent contact with the chest without excessive tightness or looseness, following manufacturer guidelines [16]. ECG leads were worn under the Prevayl garments to restrict movement of the cables and reduce motion artefacts within the ECG data. Electrode placement followed the standard Mason–Likar modified 3-lead exercise ECG configuration: one electrode below the right clavicle (with the Faros device attached), one below the left clavicle, and one on the left side of the chest below the 12th rib. For female participants, the two sub-clavicular electrodes sit above the bra band, and the inferior electrode sits below it; the ECG cable was routed beneath the Prevayl garment between these sites. The Prevayl garment, therefore, sat over the cable in the same manner as it would over any underlying layer, closely mirroring real-world use. For male participants wearing the t-shirt, the same sub-garment cable routing was applied to ensure consistency across the sample.

### 2.4. Protocol

The protocol was informed by previous validation studies [17,18] but was specifically developed to enhance methodological rigour and provide a more comprehensive evaluation of the Prevayl wearable system. Unlike earlier protocols that focused on short-duration steady-state stages, self-selected running speeds, or limited activity types, this study implemented a continuous 16-min test composed of eight two-minute stages ranging from rest to high-intensity running (4–12 kph), including both warm-up and recovery phases. This design allowed for the capture of heart rate data across a broad spectrum of intensities in real time, improving the ecological validity of the test and representing the demands of the device in practice. By continuously recording and analysing beat-to-beat data across varied speeds, rather than at isolated measurement points, this protocol more closely mirrors real-world athletic and fitness contexts in which heart rate fluctuates dynamically. As such, the study offers a novel contribution to the validation of garment-based heart rate monitors by employing a realistic, multi-intensity treadmill protocol that integrates rest, exertion, and recovery within a single continuous assessment. Participants began by resting for two minutes and standing while the treadmill was stationary (baseline) before progressing from 4 kph to 12 kph in two-minute intervals (4 kph, 6 kph, 8 kph, 10 kph, and 12 kph). Once the participant had run for two minutes at 12 kph, they reduced their speed back to 4 kph over two minutes to cool down before standing for two minutes again while the treadmill was stationary (recovery). Heart rate data were recorded simultaneously on the ECG and the Prevayl wearable throughout the test and compared retrospectively.

### 2.5. Data Processing

All data were exported from the ECG device as a CSV file containing raw ECG signals. Corresponding data from the Prevayl device were obtained via their online portal (v 2.12) and included both raw ECG signals and heart rate (bpm) calculated from the device’s proprietary algorithm. As the aim of the analysis was to compare heart rate values (beats per minute) between devices, rather than conduct detailed ECG interpretation or QRS morphology assessment, a simple peak detection method was implemented in MATLAB to identify R peaks from the raw ECG and Prevayl signals across the 16-min recording. For the reference ECG, the lead producing the highest R-wave amplitude was selected for peak detection on a participant-by-participant basis. In practice, this corresponded to the bipolar chest lead derived from the two sub-clavicular electrodes (approximating Lead I configuration) in all participants, consistent with the principle that R-wave detection should be performed on the lead offering the clearest QRS morphology [19]. This lead configuration most closely mirrors the single thoracic lead used by the Prevayl garment, supporting a like-for-like comparison. Peak detection was performed within four-second windows, and the number of detected beats was multiplied by 15 to calculate bpm for each window. Four-second windows were chosen as this was the manner in which the Prevayl heart rate was calculated using its algorithm. While advanced and validated QRS detection algorithms exist, our approach prioritised consistent application across both datasets (ECG and Prevayl) to support comparative analysis. Notably, this method was used strictly for beat counting and not for diagnosing cardiac events or assessing waveform integrity.

R-peak detection was selected as the basis for heart rate calculation, as the R-wave represents the largest amplitude deflection in the ECG signal, typically exceeding P and T wave amplitudes by a factor of 5–10 [19], making threshold-based detection robust and reliable for beat counting purposes. Prior to peak detection, the raw ECG signal was high-pass filtered using a first-order Butterworth filter with a 5 Hz cutoff frequency to remove baseline wander whilst preserving the high-frequency components of the QRS complex. Peak detection was then performed using a threshold-based algorithm applied independently to each 4-s window of both the ECG and Prevayl raw signals, identifying local maxima that exceeded a defined amplitude threshold and were separated by a minimum inter-peak distance of 100 samples (200 ms), consistent with a physiologically plausible minimum inter-beat interval. The number of detected peaks within each 4-s window was multiplied by 15 to convert to beats per minute. This approach was applied consistently across both datasets to ensure comparability and was used solely for beat counting rather than morphological analysis or arrhythmia detection. It is acknowledged that multiplying beat counts within a 4-s window by 15 is a coarse approximation of heart rate rather than a true beat-to-beat or continuously averaged measure. Because only an integer number of beats can fall within a fixed window, the resulting bpm estimates are constrained to discrete values (e.g., 60, 75, or 90 bpm for 4, 5, or 6 beats per 4 s), and window boundary alignment with the cardiac cycle can cause estimates at the individual window level to diverge from the true heart rate by up to approximately 15 bpm. This limitation applied equally to both the ECG and Prevayl signals, since the identical algorithm was used for both, supporting comparative validity. Its effect on group-level metrics is expected to be negligible given the large number of paired windows (N_win_ = 6248), as phase alignment errors average across windows. This limitation is further noted in the Limitations section.

Additionally, Prevayl provided its own bpm values via its internal algorithm for separate comparison. To avoid ambiguity, the five data streams used in analysis are defined as follows: (i) ECG beats, count of R-peaks detected by the MATLAB threshold-based algorithm applied to the raw ECG signal from the Faros reference device; (ii) Prevayl beats, count of R-peaks detected by the same MATLAB algorithm applied to the raw ECG signal exported from the Prevayl device; (iii) ECG bpm, heart rate derived from (i) by multiplying the 4-s window beat count by 15; (iv) Prevayl bpm, heart rate derived from (ii) by the same method; and (v) Prevayl algorithm bpm, heart rate calculated by Prevayl’s proprietary on-device algorithm and exported directly from the Prevayl online portal, without MATLAB processing. Comparisons (i) vs. (ii) and (iii) vs. (iv), therefore, reflect agreement between raw sensor signals processed identically; comparison (iii) vs. (v) reflects agreement between the MATLAB-derived ECG reference and the Prevayl proprietary output.

### 2.6. Synchronisation and Sample Rate

The ECG system recorded data at a sampling rate of 500 Hz, while the Prevayl device sampled at 512 Hz. As the ECG device began recording prior to the Prevayl garment in all cases, the ECG signal was first trimmed by removing the initial samples corresponding to the calculated time difference between device start times, extracted from each device’s timestamp. Following this coarse alignment, fine synchronisation was achieved by identifying the first R-peak in each signal within the first 1000 samples. The leading samples of whichever signal contained the later first peak were then removed, ensuring both datasets shared a common starting point from the first detected heartbeat. Both signals were subsequently trimmed to equal length for comparative analysis. Given the different sampling rates, each device’s data were processed independently using the consistent four-second window approach described above, rather than resampling either signal, to avoid introducing interpolation artefacts. It is acknowledged that the two systems do not share a common hardware circuit and, therefore, hardware-level synchronisation was not feasible. To ensure the most accurate possible temporal alignment, both devices were synchronised to atomic clock time prior to each data collection session (Prevayl via iPhone clock; Faros via computer clock), providing a common external time reference. Coarse alignment was then achieved using device-embedded timestamps, and fine alignment was performed by identifying the first detected R-peak in each signal, ensuring a shared cardiac event as the starting reference point. While minor residual timing discrepancies cannot be fully excluded, the use of a common clock reference, timestamp-based coarse alignment, and R-peak-based fine alignment collectively minimises systematic offset. As noted in the Limitations section, any remaining random timing errors are unlikely to have meaningfully influenced group-level results given the large number of paired windows (N_win_ = 6248). Hardware-level synchronisation between independent commercial wearable systems is generally not achievable and is not required by established wearable validation frameworks; the software-based approach employed here is consistent with methodologies used in comparable validation studies in this field.

### 2.7. Data Analysis

Data were analysed using MATLAB (Version R2022a, The MathWorks Inc., Natick, MA, USA). Each 16-min recording was divided into non-overlapping four-second windows, resulting in 240 data windows per participant. Four-second windows were chosen to match the same window period used by Prevayl’s algorithm to calculate bpm. With 26 participant datasets, a total of 6248 paired heartbeat datasets were created, allowing comparisons between ECG and Prevayl outputs.

To quantify accuracy and differences between the two systems, the following metrics were calculated (Table 2). Beat count error (%) is calculated from total raw beat counts over the full 16-min recording per participant; heart rate absolute error and heart rate percentage error are calculated per 4-s window using bpm values.

Agreement between the two systems was assessed using Bland–Altman analysis, which estimates systematic bias and the limits of agreement across all time windows and is presented here as a visual representation of bias rather than a formal inferential test. Lin’s concordance correlation coefficient (CCC) was selected as the primary measure of agreement, as it incorporates both precision and accuracy by evaluating how far observations deviate from the line of perfect concordance [20], making it more appropriate than Pearson’s r for repeated physiological measurements of this kind [20,21]. Unlike Bland–Altman analysis, the CCC does not assume independence of observations in the same way and is, therefore, the more suitable primary statistic given the repeated-measures structure of the dataset (multiple windows nested within participants). CCC values were also computed separately by test condition (N_win_ ≈ 720 per stage) to explore performance consistency across running intensities. Given the exploratory nature of these speed-stratified analyses, the results should be interpreted as descriptive rather than confirmatory.

## 3. Results

### 3.1. Accuracy—Agreement and Difference

The absolute number of raw beats recorded over 16 min from the ECG was compared to the same beats produced by Prevayl, per participant (N_part_ = 26). Over the 16-min recording, the ECG detected a mean of 2134.7 ± 281.5 beats per participant. Beat-count error (Table 2) had a median of 100.5% (range: 98.6–104.4%), indicating that Prevayl detected, on average, 0.5% more beats than ECG; individual values ranged from −1.4% to +4.4%, with the majority of participants falling within ±2% of ECG. The median, therefore, reflects a small overall positive offset together with a distribution of both over- and under-detection across individuals, rather than a systematic directional bias.

The mean absolute difference between devices, regardless of direction, was 7.1 ± 9.1 bpm over the full 16-min recording. The corresponding mean percentage error was −0.45 ± 1.2%, derived from raw beat counts across the two devices.

### 3.2. Bland–Altman Analysis

**Figure 3:**
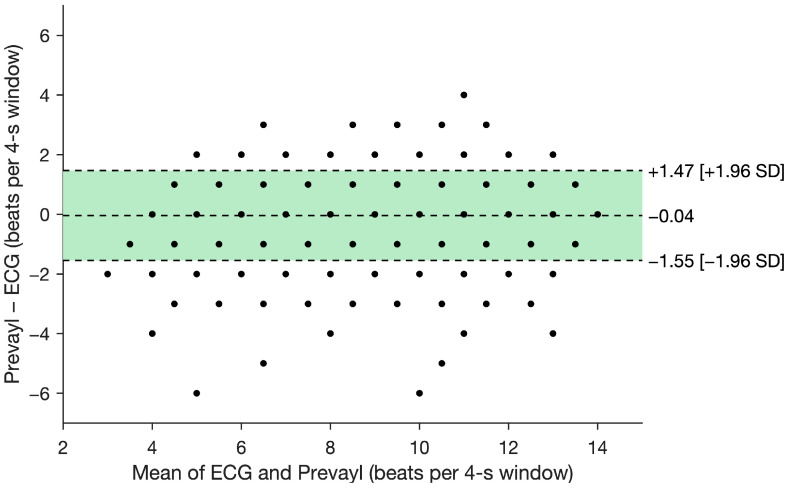
Bland–Altman plot of agreement between ECG and Prevayl raw beat counts within each 4-s window (N_win_ = 6248). The x-axis shows the mean of the two devices (beats per 4 s window); the y-axis shows the difference (ECG minus Prevayl, beats). The solid green line indicates the mean bias (0.04 beats); shaded bands represent the upper and lower 95% limits of agreement (±1.5 beats).

**Figure 4:**
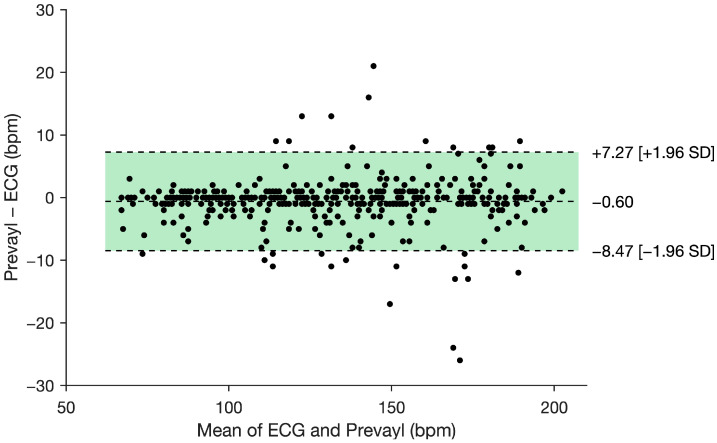
Bland–Altman plot of ECG-derived versus Prevayl-derived bpm. Solid line: mean bias (−0.6 bpm); dashed lines: 95% limits of agreement (+7.27 to −8.47 bpm). N_win_ = 411 windows, sub-sampled at approximately one window per minute per participant to reduce discretisation noise from 4 s window bpm derivation.

A Bland–Altman plot of the difference in raw beat counts between ECG and Prevayl, computed for each 4-s window across all participants (N_win_ = 6248), showed a systematic bias of 0.04 beats, with 95% of values falling within ± 1.5 beats (Figure 3). The equivalent analysis using bpm values yielded a mean bpm bias of −0.6 bpm, with limits of agreement and per-window dispersion detailed in the Limitations and Figure 4 caption.

### 3.3. Criterion Validity—Concordance Correlation Coefficient

This study found a strong correlation between raw beat count found using ECG and Prevayl (r = 0.94, *p* < 0.01) and a strong correlation between heart rate (bpm) from ECG and the Prevayl algorithm (r = 0.96, *p* < 0.01) (Table 3). At different running speeds (0–12 kph), a strong correlation was also found between raw beat count from ECG and Prevayl (r = 0.82–0.89, *p* < 0.01) and between heart rate (bpm) from ECG and Prevayl (r = 0.86–0.93, *p* < 0.01). To clarify the distinction between these two comparisons, ‘Prevayl’ in this context refers to heart rate derived from Prevayl raw ECG signals using the same MATLAB peak detection algorithm applied to the reference ECG (i.e., Prevayl bpm derived from R-peak counting); ‘Prevayl algorithm’ refers to heart rate values generated by Prevayl’s proprietary on-device processing algorithm and exported directly from the Prevayl online portal. These are distinct data streams, as detailed in Table 2 and Section 2.5. The primary aim of this study was to evaluate agreement between the Prevayl wearable system and the 3-lead ECG reference standard; comparison with the Prevayl proprietary algorithm provides a secondary and supplementary assessment of whether the device’s internal processing adds further accuracy beyond raw beat counting alone.

### 3.4. Accounting for Running Speed

When the data were stratified by running speed (Table 4), concordance correlation coefficients were lower than those for the combined dataset (Table 3), although all relationships remained statistically significant (*p* < 0.01). This reduction reflects the narrower range of heart rate values within each speed condition, which constrains the variance available to support a high correlation, rather than a true deterioration in device agreement.

## 4. Discussion

This study evaluated the accuracy and validity of the Prevayl heart rate monitor compared with a 3-lead ECG during an incremental running test, representing the first investigation to directly compare these systems. The results demonstrated a mean bpm bias of −0.6 bpm and an absolute error of 7.1 ± 9.1 bpm over the 16-min treadmill protocol. This study found a strong correlation between heart rate data (raw beat count and bpm) derived from ECG and Prevayl wearables (r = 0.94, 95% CI (0.94–0.95)), consistent with previous validation studies where chest-worn heart rate devices verified against ECG have achieved a correlation coefficient of r > 0.90 and a standard error of estimate within 5 beats per min of an ECG during rest, recovery, and varying exercise intensities [8].

The observed CCC values of 0.94 and 0.96 indicate moderate to substantial agreement according to established criteria [20], supporting the validity of the Prevayl system. The correlation remained high when the analysis was adjusted for different running speeds (0–12 kph). In addition, Bland–Altman plots demonstrated minimal systematic bias, with 95% of the data falling within narrow confidence intervals, further supporting the validity of the Prevayl system for heart rate monitoring during incremental exercise. These findings represent the first investigation to validate Prevayl garments against ECG as the criterion standard. Furthermore, this research is novel given the lack of data in garment-based devices, tested across incremental running speeds [12] to reflect their use in practice. This research, therefore, adds to the evolving evidence base examining the reliability of wearable heart rate monitors and provides important findings for users seeking to ensure accurate data collection using Prevayl garments.

The median beat count agreement of 100.5% indicates that the Prevayl device detected marginally more beats than the reference ECG on average. While individual participants showed both over- and under-detection (range −1.4% to +4.4%), the slight positive skew warrants consideration. Several mechanisms may explain this tendency. Textile-based dry electrodes operating through fabric compression can exhibit signal morphology differences compared to gel electrode systems, potentially producing additional amplitude peaks that do not correspond to true R-waves. The Prevayl device samples at 512 Hz versus 500 Hz for the reference ECG; although both signals were processed identically using the same threshold-based peak detection algorithm, minor differences in signal amplitude and resolution could influence the count of peaks exceeding the threshold within any given 4-s window. Additionally, motion artefact at higher running speeds may introduce transient deflections that are erroneously detected as R-peaks. The marginal magnitude of the overcount (0.5% on average) suggests these effects are small and unlikely to affect practical heart rate monitoring at the group level; however, users requiring single-beat precision should note this tendency. This finding aligns with previous observations that garment-based systems can exhibit occasional false-positive peak detection during dynamic activity [21].

Previous studies investigating biometric garments such as the Hexoskin biometric shirt and Astroskin biometric shirt have reported strong agreement with criterion measures during rest and steady-state exercise, with correlations often exceeding r = 0.8–0.9 and relatively small error margins when garment fit and electrode contact are optimised [17,22,23]. However, validity appears highly context-dependent, with accuracy deteriorating as movement intensity and complexity increase. For example, during repeated 400m shuttle runs, the Hexoskin garment produced erroneous heart rate data in a substantial proportion of participants at peak intensities, likely due to motion artefact and sensor displacement [24]. Similar trends are evident in other garment-based systems, in which validity declines during running compared with rest or walking (e.g., Adidas smart sports bra ICC = 0.155; Sensoria biometric bra ICC = 0.465), although some garments demonstrate excellent agreement under comparable conditions (e.g., Berlei sports bra ICC = 0.972) [14].

Data from the present study demonstrate that Prevayl garments provide consistently strong agreement with the criterion measure across conditions, with correlations of r = 0.85 (raw beat value) and 0.86 (Prevayl algorithm) at rest, r = 0.82–0.90 during running at 6–12 kph, and r = 0.89–0.93 during recovery. While a reduction in validity was observed with increasing running speed, consistent with the previous literature, correlations during running remained within an acceptable range, suggesting resilience to motion artefact during incremental treadmill exercise. Overall agreement across conditions (r = 0.94 for raw beat values; r = 0.96 for the Prevayl algorithm) exceeded that reported for several wrist-worn devices shown in previous work [17,18] for Apple (r = 0.91, 0.93), Mio (r = 0.91), Fitbit (r = 0.76, 0.84), Basis Peak (r = 0.83), Garmin (r = 0.92), and TomTom (r = 0.88) but was lower than Polar devices (r = 0.99). In addition, Prevayl had a stronger correlation coefficient than the Adidas smart sports bra (ICC: 0.447) and the Sensoria biometric bra (0.625) but was lower than the Berlei sports bra (0.978) [14] during rest, walking, and running.

Using a Bland–Altman analysis, Wang et al. [18] found that the Apple Watch and Mio Fuse had 95% of differences fall within −27 bpm and +29 bpm of the electrocardiogram, while the Fitbit Charge heart rate had 95% of values within −34 bpm and +39 bpm, and the corresponding values for the Basis Peak were within −39 bpm and +33 bpm. Gillinov et al. [17], using Bland–Altman analysis, revealed that the Apple Watch had 95% of differences fall within −17 and 20 bpm of the ECG, whereas TomTom Spark Cardio and Garmin Forerunner 235 had 95% of values fall within −24 and 31 bpm and −27 and 33 bpm, respectively. The present study shows that 95% of differences from the Prevayl data are within −8.47 to +7.27 bpm, which presents a smaller range than those shown by all brands tested in Wang et al. [18] and Gillinov et al. [17]. These findings are important for athletes and professionals seeking accurate heart rate data collection during physical activity so that they can be sure that their performance monitoring is reliable. The absolute error of 7.1 ± 9.1 bpm should be contextualised within the sports wearable literature rather than against clinical ECG device standards, which were developed for diagnostic equipment and are not applicable to consumer heart rate monitors. The limits of agreement reported here compare favourably with those reported for wrist-worn devices under equivalent exercise conditions [17,18], supporting the suitability of the Prevayl system for athletic and recreational heart rate monitoring.

These findings align with the previous literature showing high validity under resting and recovery conditions but extend existing evidence by demonstrating robust validity during an incremental running test, a condition that remains underrepresented in the existing research [3,10], despite its relevance to applied monitoring. While this may suggest improved signal stability during continuous locomotion, the broader literature highlights the susceptibility of textile-based ECG systems to motion artefact during more dynamic or high-intensity activity [24]. Comparisons with previous work should, however, be made with caution due to the methodological heterogeneity, including differences in exercise modality (cycling vs. running), protocol design (self-selected vs. prescribed intensity), and the limited availability of data from incremental running tests. For example, when on the treadmill, Gillinov et al. [17] collected heart rate data on completion of three 90-s stages and at the end of each two-minute rest period between exercises. This led to a smaller sample size comparison (range 75–150, based on device), whereas the present study included 6248 windows and roughly N_win_ ≈ 720 when divided by running speed. Accordingly, further research is required to establish the ecological validity of Prevayl garments in sport-specific and field-based conditions.

### Limitations

The primary aim of this study was to evaluate the validity of the device during incremental running, as this represents one of the most common and practically relevant cases for wearable heart rate monitoring [25]. Nonetheless, device performance may vary across different movement patterns and exercise modalities [17], and, therefore, this is an important avenue for future research to increase the generalisability of the findings to other forms of physical activity.

Despite efforts to synchronise both devices using atomic clock references, minor timing discrepancies may have occurred during the manual start and stop processes. These errors are expected to be random rather than systematic and are unlikely to have meaningfully influenced the results given the sample size and analytical approach. Similarly, occasional inconsistencies in peak detection were observed during data processing, most likely attributable to lower-amplitude R-peaks falling below the detection threshold. Because the R-wave is the dominant feature of the ECG signal, threshold-based detection is generally robust, but occasional missed beats cannot be excluded, particularly at higher exercise intensities where motion artefact may reduce signal amplitude. No manual correction was applied, and the strong correlations observed indicate that any missed peaks did not meaningfully affect the outcomes; if anything, these omissions are likely to represent a conservative estimate of agreement. Across 6248 paired windows, the effect of any such random errors is expected to be negligible at the group level.

ECG leads were routed beneath the Prevayl garment to minimise cable movement artefact in the reference signal; while this arrangement closely mirrors real-world layering conditions, it may not perfectly replicate fully independent use of each system. This setup may have reduced observable discrepancies between devices by stabilising the ECG signal and should be considered when interpreting the agreement metrics reported. The repeated-measures structure of the dataset, comprising multiple 4-s windows nested within participants, means that observations are not fully independent. The CCC was selected as the primary agreement metric in part because of its suitability for this data structure; however, readers should bear this in mind when interpreting the Bland–Altman limits of agreement, which assume independent observations.

A further consideration relates to the spatial relationship between the reference ECG electrodes and the Prevayl sensing surfaces during simultaneous wear. The 3-lead ECG configuration uses three discrete adhesive electrodes placed at fixed thoracic landmarks, whereas the Prevayl garment incorporates conductive textile electrodes distributed laterally around the chest band rather than confined to discrete points. Where an ECG sticker coincided with a Prevayl textile electrode, localised occlusion of that small patch of textile–skin contact was unavoidable. However, because the Prevayl electrode area is distributed around the thorax, the occluded regions represented only a small fraction of the total active contact surface, and the device retained substantial uninterrupted textile–skin contact elsewhere around the band from which to acquire signal. The opposite concern, that routing the ECG cables beneath the garment may have stabilised the reference signal in a way that does not reflect independent ECG use, has been noted earlier in this section. The net direction of any resulting bias is, therefore, not straightforward to predict. Furthermore, the layered configuration tested here mirrors typical real-world use, in which the Prevayl garment is commonly worn over a base layer or sports bra. Garment fit was verified for each participant following manufacturer guidelines to ensure consistent textile electrode contact pressure across the thoracic region. A counterbalanced design comparing Prevayl-alone versus Prevayl-over-ECG conditions in the same participants would directly quantify any contact interference effect and represents a useful direction for future work.

The use of 4-s windows to derive bpm by multiplying beat counts by 15 represents a coarse approximation that introduces inherent quantisation error at the level of individual windows. Because only an integer number of beats can be detected within a fixed window, the resulting bpm estimates take discrete values, and window boundary phase alignment with the cardiac cycle can produce estimates that diverge from the true heart rate by up to approximately 15 bpm at the single-window level. This limitation applied equally to both the ECG and Prevayl signals; its effect on group-level agreement metrics is expected to be negligible across the large number of paired windows analysed. Nonetheless, the results from individual 4-s windows should be interpreted as approximations of average heart rate within that interval rather than as precise instantaneous measures.

Furthermore, this study was conducted at a single site with a relatively homogeneous sample of young, healthy, active adults in controlled laboratory conditions, which may limit generalisability to older adults, clinical populations, or outdoor running environments.

## 5. Conclusions

The Prevayl system demonstrates reliable estimation of average heart rate (within 4-s windows) when compared to a 3-lead ECG during an incremental treadmill running test. Strong concordance correlations (r = 0.94 for raw beat count; r = 0.96 for the Prevayl proprietary algorithm) were observed between ECG and Prevayl-derived heart rate data across the full 16-min recording. Speed-stratified analyses confirmed that correlations remained within acceptable limits across all test conditions (r = 0.82–0.93), and Bland–Altman analysis revealed minimal systematic bias, with 95% of values falling within narrow limits of agreement. Furthermore, the Prevayl algorithm enhanced the accuracy of heart rate estimation beyond raw signal processing alone, yielding higher concordance with ECG bpm than the MATLAB-derived beat count comparator. These findings support the use of Prevayl garments as a reliable tool for heart rate monitoring during exercise and offer practical value for athletes, coaches, and practitioners seeking accurate, non-invasive monitoring of cardiovascular responses during exercise.

## Figures and Tables

**Table 1:** Participant demographics.

	Mean ± SD	Range
Age, years	32.0 ± 10.4	19–58
Gender	15 female; 11 male	
Height, cm	170.3 ± 9.6	157–188
Weight, kg	70.1 ± 12.4	53–101

**Table 2:** Equations for accuracy data analysis.

Measure	Equation
Beat count error (%)	(Prevayl beats/ECG beats) × 100
Heart rate absolute error (bpm)	|ECG bpm − Prevayl bpm|
Heart rate percentage error (%)	(Prevayl bpm − ECG bpm)/ECG bpm × 100

**Table 3:** Overall correlation between ECG and Prevayl with 95% confidence intervals for the 16-min recording. All results were significant (*p* < 0.01). *Note* that the results between beats and bpm are the same due to the same underlying data (beats) being used, where bpm is calculated from this.

	Prevayl Beats *r* (95% CI)	Prevayl bpm *r* (95% CI)	Prevayl Algorithm *r* (95% CI)
ECG beats	0.94 (0.94–0.95)		
ECG bpm		0.94 (0.94–0.95)	0.96 (0.96–0.96)

**Table 4:** Concordance correlation coefficient at each speed with 95% confidence intervals between ECG beats and Prevayl beats. All results demonstrated significance (*p* < 0.01).

Speed	ECG bpm vs. Prevayl bpm	ECG bpm vs. Prevayl Algorithm
Baseline (resting)	0.85 (0.83–0.87)	0.86 (0.84–0.88)
4 kph	0.84 (0.82–0.86)	0.85 (0.83–0.87)
6 kph	0.88 (0.86–0.89)	0.90 (0.89–0.91)
8 kph	0.85 (0.83–0.87)	0.90 (0.89–0.91)
10 kph	0.82 (0.79–0.84)	0.89 (0.87–0.90)
12 kph	0.83 (0.81–0.85)	0.88 (0.86–0.90)
Cool down	0.89 (0.87–0.90)	0.93 (0.93–0.94)
Baseline (recovery)	0.89 (0.87–0.90)	0.93 (0.93–0.94)

## Data Availability

The original contributions presented in this study are included in the article/Supplementary Materials. Further inquiries can be directed to the corresponding author.

## Supplementary Materials

The following supporting information can be downloaded at: https://www.mdpi.com/article/10.3390/s26113378/s1.

## References

[1] Thompson W.R. (2022). Worldwide survey of fitness trends for 2022. ACSMS Health Fit. J..

[2] Spierer D.K., Rosen Z., Litman L.L., Fujii K. (2015). Validation of photoplethysmography as a method to detect heart rate during rest and exercise. J. Med. Eng. Technol..

[3] Machado A., Ferreira D.F., Ferreira S., Almeida-Antunes N., Carvalho P., Melo P., Rocha N., Rodrigues M.A. (2025). A Systematic Review of Chest-Worn Sensors in Cardiac Assessment: Technologies, Advantages, and Limitations. Sensors.

[4] Merrigan J.J., Stovall J.H., Stone J.D., Stephenson M., Finomore V.S., Hagem J.A. (2022). Validation of Garmin and Polar Devices for Continuous Heart Rate Monitoring During Common Training Movements in Tactical Populations. Meas. Phys. Educ. Exerc. Sci..

[5] Dudarev V., Barral O., Zhang C., Davis G., Enns J.T. (2023). On the Reliability of Wearable Technology: A Tutorial on Measuring Heart Rate and Heart Rate Variability in the Wild. Sensors.

[6] Stracina T., Ronzhina M., Redina R., Novakova M. (2022). Golden Standard or Obsolete Method? Review of ECG Applications in Clinical and Experimental Context. Front. Physiol..

[7] Weiler D.T., Villajuan S.O., Edkins L., Cleary S., Saleem J.J. (2017). Wearable heart rate monitor technology accuracy in research: A comparative study between PPG and ECG technology. Proc. Hum. Factors Ergon. Soc. Annu. Meet..

[8] Engström E., Ottosson E., Wohlfart B., Grundström N., Wisén A. (2012). Comparison of heart rate measured by Polar RS400 and ECG, validity and repeatability. Adv. Physiother..

[9] Etiwy M., Akhrass Z., Gillinov L., Alashi A., Wang R., Blackburn G., Gillinov S.M., Phelan D., Gillinov A.M., Houghtaling P.L. (2019). Accuracy of wearable heart rate monitors in cardiac rehabilitation. Cardiovasc. Diagn. Ther..

[10] Carrier B., Barrios B., Jolley B.D., Navalta J.W. (2020). Validity and Reliability of Physiological Data in Applied Settings Measured by Wearable Technology: A Rapid Systematic Review. Technologies.

[11] Yang K., McErlain-Naylor S.A., Isaia B., Callaway A., Beeby S. (2024). E-Textiles for Sports and Fitness Sensing: Current State, Challenges, and Future Opportunities. Sensors.

[12] Khundaqji H., Hing W., Furness J., Climstein M. (2020). Smart Shirts for Monitoring Physiological Parameters: Scoping Review. JMIR mHealth uHealth.

[13] Smith C.M., Chillrud S.N., Jack D.W., Kinney P., Yang Q., Layton A.M. (2019). Laboratory Validation of Hexoskin Biometric Shirt at Rest, Submaximal Exercise, and Maximal Exercise While Riding a Stationary Bicycle. J. Occup. Environ. Med..

[14] Navalta J.W., Ramirez G.G., Maxwell C., Radzak K.N., McGinnis G.R. (2020). Validity and Reliability of Three Commercially Available Smart Sports Bras during Treadmill Walking and Running. Sci. Rep..

[15] Peake J.M., Kerr G., Sullivan J.P. (2018). A Critical Review of Consumer Wearables, Mobile Applications, and Equipment for Providing Biofeedback, Monitoring Stress, and Sleep in Physically Active Populations. Front. Physiol..

[16] Limited P. Prevayl Innovations. https://www.prevayl.com/.

[17] Gillinov S., Etiwy M., Wang R., Blackburn G., Phelan D., Gillinov A.M., Houghtaling P., Javadikasgari H., Desai M.Y. (2017). Variable Accuracy of Wearable Heart Rate Monitors during Aerobic Exercise. Med. Sci. Sports Exerc..

[18] Wang R., Blackburn G., Desai M., Phelan D., Gillinov L., Houghtaling P., Gillinov M. (2017). Accuracy of Wrist-Worn Heart Rate Monitors. JAMA Cardiol..

[19] Kohler B.U., Hennig C., Orglmeister R. (2002). The principles of software QRS detection. IEEE Eng. Med. Biol. Mag..

[20] McBride G.B. (2005). A Proposal for Strength-of-Agreement Criteria for Lin’s Concordance CORRELATION Coefficient.

[21] Tsukada Y.T., Tokita M., Murata H., Hirasawa Y., Yodogawa K., Iwasaki Y.K., Asai K., Shimizu W., Kasai N., Nakashima H. (2019). Validation of wearable textile electrodes for ECG monitoring. Heart Vessel..

[22] Elliot C.A., Hamlin M.J., Lizamore C.A. (2019). Validity and Reliability of the Hexoskin Wearable Biometric Vest During Maximal Aerobic Power Testing in Elite Cyclists. J. Strength Cond. Res..

[23] Macquarrie A., Sidhu J., Deetlefs C., Whitfield S., Stainer M. (2024). Establishing the Validity and Reliability of the Astroskin^®^ Biometric Shirt. Top. Exerc. Sci. Kinesiol..

[24] Haddad M., Hermassi S., Aganovic Z., Dalansi F., Kharbach M., Mohamed A.O., Bibi K.W. (2020). Ecological Validation and Reliability of Hexoskin Wearable Body Metrics Tool in Measuring Pre-exercise and Peak Heart Rate During Shuttle Run Test in Professional Handball Players. Front. Physiol..

[25] Achten J., Jeukendrup A.E. (2003). Heart rate monitoring: Applications and limitations. Sports Med..

